# Health Behaviours during Pregnancy in Women with Very Severe Obesity

**DOI:** 10.3390/nu7105403

**Published:** 2015-10-07

**Authors:** Nor A. Mohd-Shukri, Andrew Duncan, Fiona C. Denison, Shareen Forbes, Brian R. Walker, Jane E. Norman, Rebecca M. Reynolds

**Affiliations:** 1 Endocrinology Unit, University/BHF Centre for Cardiovascular Science, Queen’s Medical Research Institute, University of Edinburgh, Edinburgh EH16 4TJ, UK; norazwani@iium.edu.my (N.A.M.-S.); shareen.forbes@ed.ac.uk (S.F.); b.walker@ed.ac.uk (B.R.W.); 2 Scottish Trace Element and Micronutrient Reference Laboratory, Macewen Building, Royal Infirmary, Glasgow G4 0SF, UK; andrew.duncan@ggc.scot.nhs.uk; 3 Tommy’s Centre for Maternal and Fetal Health, MRC Centre for Reproductive Health, Queen’s Medical Research Institute, University of Edinburgh, Edinburgh EH16 4TJ), UK; Fiona.denison@ed.ac.uk (F.C.D.); jane.norman@ed.ac.uk (J.E.N.)

**Keywords:** obesity, pregnancy, diet, exercise, lifestyle, micronutrients

## Abstract

The health behaviours of pregnant women with very severe obesity are not known, though these women are at high risk of pregnancy complications. We carried out a prospective case-control study including 148 very severely obese (BMI >40 kg/m^2^) and 93 lean (BMI <25 kg/m^2^) pregnant women. Diet, physical activity, smoking, alcohol and folic acid consumption were assessed by questionnaire in early and late (16 and 28 weeks gestation) pregnancy. Circulating levels of iron, vitamin B12 and folate and other essential trace elements and minerals were measured in a subset at each time point. The findings biochemically confirmed that very severely obese women consumed diets that were energy-rich but poor in essential micronutrients. A third of all women met physical activity recommendations for pregnancy. A third of very severely obese women and two thirds of lean women took folic acid supplements prior to pregnancy. Very severely obese women were more likely to smoke but less likely to drink alcohol than lean women (all *p* < 0.05). Women with very severe obesity have low self-reported intakes and circulating levels of essential micronutrients in pregnancy and few follow current recommendations for pregnancy nutrition and lifestyle. These high-risk women represent a group to target for education about health behaviours prior to and during pregnancy.

## 1. Introduction

The prevalence of obesity among women of reproductive age has risen dramatically in the last two decades, with one in five women in the UK now obese at antenatal booking [1]. Rates of very severe obesity (Class III obesity, body mass index (BMI) ≥40 kg/m^2^) have also increased, recently estimated as 6.5% of women of childbearing age in the USA [2]. Obesity in pregnancy is one of the most challenging current obstetric problems and is associated with adverse pregnancy outcomes for both mother and child [3,4]. 

A woman’s nutrition, exercise and health behaviours during pregnancy have important implications for pregnancy outcome [5]. Although information about recommended health behaviours during pregnancy is widely available for all women, there is little specific advice for obese women other than to limit gestational weight gain to 5–9 kg [2] and to take a higher dose (5 mg) of folic acid [6]. Further, many obese women are unaware of the health risks associated with obesity in pregnancy, and so may not consider altering lifestyle behaviours during pregnancy. There is some evidence that overweight and obese women consume diets of poorer quality than normal-weight women in early pregnancy [7,8,9,10] but it is not known whether diet and other health behaviours of women with more severe obesity differ during pregnancy from that of lean women. Such knowledge would be useful to inform development of specific guidance for very severely obese pregnant women planning pregnancy. Arguably, very severely obese women would potentially benefit most from lifestyle intervention. As it is known that dietary behaviours in normal-weight women differ little during pregnancy compared with prior to pregnancy [11], we hypothesised that women with very severe obesity have a poorer diet and do less exercise during pregnancy than lean women and are less adherent to other lifestyle recommendations for a healthy pregnancy. To test our hypothesis we studied diet and lifestyle behaviours during pregnancy in a high-risk group of pregnant women with very severe obesity compared to normal-weight controls. 

## 2. Experimental Section

### 2.1. Subjects

The study participants included 148 very severely obese (antenatal booking BMI >40 kg/m^2^) and 93 lean women (antenatal booking BMI <25 kg/m^2^), with a singleton pregnancy, attending an antenatal metabolic clinic at the Royal Infirmary, Edinburgh, UK, and participating in a larger study examining the consequences of very severely obese pregnancy. Details of the overall cohort have been previously described [12,13]. Briefly, very severely obese women were referred to the clinic for specialist care by their community midwives after their antenatal booking appointment. Lean women were recruited into the study from community antenatal clinics. The subset of women included in this study were the first 241 participants recruited to the overall study and there were no differences in their demographics from the overall cohort. All very severely obese women were reviewed by a dietician in early pregnancy and given advice about healthy eating. The dietician did not routinely review lean women but was available as a referral resource if the clinician or woman had any concerns. Ethical approval was obtained from the Lothian Research Ethics committee and all participants gave informed written consent (REC reference number 08/S1101/39). 

### 2.2. Clinical and Biochemical Measurements

Following recruitment, subjects completed a baseline questionnaire describing demographics including age, parity, social class according to Deprivation Category Score (DEPCAT, which divides postcode sectors into categories in relation to income, employment, health, housing and education) [14], and lifestyle choices including folic acid intake prior to conception and during the first 12 weeks of pregnancy, alcohol intake and smoking status. Subjects attended a study visit during early pregnancy (–16 weeks gestation) and late pregnancy (–28 weeks gestation) after an overnight fast. A venous blood sample was taken and serum and plasma frozen at −80 °C, and after a light refreshment, participants completed questionnaires regarding food intake and physical activity during early and late pregnancy. 

Food and nutrient intake was assessed using the semi-quantitative Scottish collaborative group food frequency questionnaire (SCG-FFQ) version 6.6 (Scottish Collaborative Group, University of Aberdeen, UK) [13], with measurement of serum micronutrient levels in a random subset (*n* = 25) of women. The SCQ-FFQ consists of 170 foods and drinks, divided into 21 groups and was used to assess the frequency and quantity of food intake over the preceding 2–3 months. It has been validated in both healthy adults and very severely obese pregnant women [15]. Macro and micro-nutrient intake was extracted from questionnaires as previously described [16]. Dietary patterns were assessed using principal component analysis of the different food groups [11]. 

Physical activity during pregnancy was measured using a validated pregnancy physical activity questionnaire (PPAQ) [17]. The PPAQ assesses the frequency, duration and intensity of activity during pregnancy and contains 36 questions regarding time spent in 32 activities (such as sedentary, household/care-giving, occupational and sporting activities). 

### 2.3. Laboratory Analyses

Circulating levels of a number of important vitamins and trace elements were measured in a random subset of paired samples from early and late gestation (*n* = 25 per group). Serum levels of iron, ferritin, folate and vitamin B12 were measured using an automated analyser (Architect C-16000 or Architect I-2000, Abbott Diagnostics Ltd., Maidenhead, Berks, UK, SL6 4XF) in the Biochemistry Laboratories, Royal Infirmary of Edinburgh, Edinburgh, UK. Plasma samples were used to measure levels of vitamin A, vitamin E and cholesterol by high performance liquid chromatography; copper, zinc, and selenium by inductively coupled plasma mass spectrometry (Agilent Technologies, Stockport, Cheshire, UK SK8 3GR); and 25 hydroxyvitamin D (25(OH)D) by tandem mass spectrometry (Waters Acquity; UPLC and TQD, Hertfordshire, UK WD6 3SZ). Analyses were conducted at the Scottish Trace Element and Micronutrient Reference Laboratory, Glasgow, UK. CRP and albumin were measured using an automated analyser (Architect; Abbot Diagnostics Ltd., Maidenhead, Berks, UK, SL6 4XF) to aid interpretation of the zinc, selenium and copper concentrations which alter with degree of inflammatory response [18].

### 2.4. Statistical Analyses

Normal distribution of data was assessed visually using histograms. Self-reported total energy, nutrient intakes and physical activity data were not normally distributed and were normalized by ln-transformation. Nutrient intakes were adjusted for total energy intake by using the residual method calculated from regression of nutrient intake as the dependent variable and total energy as the independent variable [19]. Nutrient intakes from dietary supplements were not included in any of the analyses. Comparisons of nutrition and physical activity within groups (obese or lean) used paired *t*-tests and between groups used independent *t*-tests. Comparisons for categorical data used chi-squared tests. Multiple regression was used to adjust for confounding factors including age, parity, ethnicity, DEPCAT status, smoking and employment status. Eating patterns were assessed using principal component analysis. A component score was calculated by multiplying the factor loading by the corresponding standardized value (*Z*-score) for each food group identified from the questionnaires (bread, milk, eggs, fish *etc.*). Each component derived provides summary coefficients for each food group with the higher score indicating greater loading of the food group within the component. All statistical analyses were performed using SPSS version 19 (SPSS Inc., Chicago, IL, USA). Significance was accepted at *p* < 0.05.

## 3. Results 

### 3.1. Subject Characteristics 

Table 1 shows the characteristics of the study participants. Very severely obese women were significantly younger, of higher parity and lower socio-economic status than the lean women. 

### 3.2. Diet and Physical Activity in Pregnancy

Table 2 shows that in early pregnancy, very severely obese women reported significantly higher intake of total energy and macronutrients (protein, fat, carbohydrates) in comparison with lean women in univariate analysis, but these findings were no longer significant after adjustment for demographics and total energy intake. Very severely obese women also reported significantly lower intake of essential micronutrients compared to lean women (Table 2). The lower reported intakes of iron, folate, vitamins D, E and K, magnesium, calcium, zinc, manganese, riboflavin and biotin remained significant following adjustment for demographics and total energy intake. The differences in intake of these essential micronutrients between the obese and lean group persisted in late gestation (Table 2). 

**Table 1:** Characteristics of study participants.

Characteristic	Lean (*n* = 93)	Very severely obese (*n *= 148)	*p*-value
	Mean (SD)	Mean (SD)	
	or *n* (%)	or *n* (%)	
BMI (kg/m^2^)	22.6 (1.7)	43.9 (4.3)	<0.0001
Age (years)	33.7 (4.2)	31.5 (5.4)	0.002
Parity			
Nulliparous	57 (64)	84 (48)	<0.001
Deprivation Category			
Least deprived	25 (29)	14 (8)	<0.001
Most deprived	60 (69)	135 (77)	?
Ethnicity			
Caucasian	93 (100)	144 (97)	0.127
Smoking Status			
Never smoked	48 (53)	82 (59)	0.004
Ex-smoker	27 (30)	16 (12)	?
Given up in last 12 months or for pregnancy	12 (13)	24 (18)	?
Still Smoking	4 (4)	15 (11)	?
Alcohol Intake (drinks/week)			
0	12 (14)	47 (36)	<0.0001
1	13 (15)	26 (20)	?
2	57 (65)	55 (42)	?
3 or more	6 (6)	3 (2)	?
Folic Acid Intake			<0.0001
Prior to Conception	55 (60)	54 (31)	?
<12 weeks gestation	34 (38)	114 (66)	?
>12 weeks gestation	2 (2)	3 (1)	?
None	0 (0)	5 (2)	?
Folic Acid Dose			
400 µg	84 (98%)	129 (96%)	0.56
5 mg	2 (2%)	5 (4%)	?

Analysis of serum levels of micronutrients in the random subset of 25 very severely obese compared with 25 lean women confirmed the significantly lower circulating levels of iron, vitamin B_12_, folate, vitamin A, copper, zinc and selenium with trends for lower Vitamin E/cholesterol and 25(OH)D in very severely obese in early pregnancy (Table 3). Exclusion of subjects with high CRP results attenuated the selenium findings and the zinc/albumin ratio did not differ between very severely obese and lean. There were similar patterns in late pregnancy other than for iron, vitamin A, zinc and selenium which were no longer significantly different between groups.

**Table 2:** Reported daily nutrient intakes in lean and very severely obese women in early and late pregnancy.

Nutrient	Early pregnancy						Late pregnancy					
	Lean *n* = 93		Obese *n *= 148				Lean *n* = 87		Obese *n *= 146			
	Median	C^25^–C^75^	Median	C^25^–C^75^	*P*	*P*^adj^	Median	C^25^–C^75^	Median	C^25^–C^75^	*P*	*P*^adj^
Energy (kcal)	2349	(1995–2846)	2435	(1975–3040)	*		2354	(1957–2876)	2173	(1747–2672)	*	
Protein (g)	94.1	(75–114)	98.6	(73–125)	*		90.5	(76–117)	87.7	(69–113)	*	
Fat (g)	91.6	(75–118)	95.6	(73–122)	*		91.8	(73–124)	87.9	(65–108)		
Carbohydrate (g)	302.1	(252–355)	309.8	(249–387)	*		304.1	(258–361)	278.8	(227–346)	*	
SFA (g)	34.6	(28–28)	38.5	(28–51)	*		36.2	(29–50)	35.1	(25–44)		
MUFA (g)	32.1	(26–42)	33.2	(25–43)	*		31.9	(25–42)	30.1	(22–37)	*	
PUFA (mg)	15.9	(13–20)	16	(12–22)	**		15.2	(13–20)	14.1	(11–19)	*	
Cholesterol (g)	277	(191–353)	290.5	(202–390)			255	(189–365)	271.5	(197–376)		
Sugars (g)	134.9	(109–176)	150.3	(113–195)	*		142	(110–179)	132.7	(99–174)	**	
Starch (g)	150	(130–196)	156	(121–191)	*		158	(121–183)	136	(107–175)	*	
Fibre (g)	22.8	(20–29)	20.6	(16–26)	*	**	23.3	(18–27)	19	(14–24)	**	*
Sodium (mg)	3200	(2375–3969)	3389	(2551–4065)	*		3239	(2638–3896)	2926	(2298–3595)		
Potassium (mg)	3876	(3262–4679)	3953	3036–5017)	*		3813	(3265–4769)	3476	2845–4648	*	
Calcium (mg)	1233	(1006–1496)	1195.5	(945–1603)	*	*	1268	(1015–1579)	1077	(837–1415)		*
Magnesium (mg)	426	(375–519)	399.5	(327–487)	*	**	417	(355–527)	359	(289–452)	*	**
Phosphorus (mg)	1791	(1483–2106)	1779.5	(1423–2311)	*		1753	(1476–2215)	1652	(1276–2097)	*	*
Iron (mg)	16.5	(14–20)	14.5	(11–19)	*	**	16.1	(13–20)	13	(10–17)	*	**
Copper (mg)	3.2	(2–4)	2.9	(2–4)	*		3.1	(2.7–4)	2.6	(2.1–3.4)		**
Zinc (mg)	12.1	(9–15)	12.1	(9–15)	*	*	11.8	(9–15)	10.9	(8–14)	*	
Chloride (mg)	4786	(3899–6097)	4998	(3873–6273)	*		4871	(3992–5900)	4428.5	(3552–5523)		
Manganese (mg)	4.6	(4–6)	3.6	(3–5)	*	**	4.5	(3–6)	3.2	(2.6–4.3)	*	**
Selenium (µg)	64	(51–80)	61	(44–84)	**	*	63	(51–80)	58	(41–77)	**	
Iodine (µg)	233	(186–307)	246.5	(173–313)			249	(189–331)	221.5	(157–290)	*	*
Retinol (µg)	320	(228–436)	319	(220–466)			316	(221–504)	290.5	(200–423)		
β–carotene (µg)	4362	(3081–6848)	4109	(2361–6127)		*	4082	(2594–6533)	3340	(2245–5023)		
Vitamin D (µg)	3.8	(2.8–5.7)	3.1	(2–4.6)		*	4	(2.7–5.7)	2.8	(1.9–4.4)		*
Vitamin E (mg)	12	(9.8–15)	11.4	(8.3–13.4)	*	*	12	(9–15.6)	10.2	(7–13)	**	*
Thiamine (mg)	2	(1.7–2.3)	2	(1.6–2.5)	*		2	(1.6–2.4)	1.8	(1.5–2.3)	*	
Riboflavin (mg)	2.1	(1.8–2.7)	2.2	(1.5–2.8)	**	*	2.2	(1.8–2.9)	1.9	(1.4–2.6)	*	**
Niacin (mg)	23.2	(19–28)	24.5	(18–31)	**		22.5	(18–28)	22.6	(16–29)	*	
Potential niacin (mg)	20.9	(16–25)	20.5	(15–27)	*		19.4	(16–26)	18.4	(14–24)	*	*
Vitamin B6 (mg)	2.5	(2–3)	2.7	(2–3.3)	*		2.5	(2.1–3.2)	2.5	(1.8–3.1)	*	
Vitamin B12 (mg)	6.2	(4.6–8.8)	6.2	(4.2–8.6)			6	(4.9–9)	5.7	(3.9–7.7)		
Folate (µg)	350	(282–439)	310.5	(225–387)	*	**	362	(268–436)	276	(202–353)	*	**
PA (mg)	6.2	(5.1–7.7)	6.5	(4.8–8.1)	*		6.2	(5.1–7.9)	5.7	(4.5–7.7)	*	
Biotin (µg)	45.4	(37–56)	41.8	(31–52)	*	**	44.1	(37–59)	37.2	(27–50)	**	**
Vitamin C (mg)	160	(120–204)	160	(110–227)	**		151	(111–102)	147	(86–199)	**	
Vitamin K (mg)	69.9	(43–137)	34	(22–68)		**	57.3	(39–132)	32.6	(17–67)		**

C^25^: 25th Percentile, C^75^: 75th Percentile; *P*: Ln-transformed crude intakes; *P*^adj^: Ln-transformed crude intakes adjusted for total energy intake, age, parity, ethnicity & DEPCAT; * *p* < 0.05; ** *p* < 0.001.

**Table 3:** Serum levels of essential micronutrients in early and late pregnancy.

	Early pregnancy (16 weeks)			Late pregnancy (28 weeks)		
	Obese *n *= 25	Lean *n *= 25	p *	Obese *n* = 25	Lean *n *= 25	p *
Iron (μmol/L)	15.7 (4.7)	23.4 (5.9)	<0.0001	12.5 (5.3)	14.7 (7.1)	0.24
Vitamin B12 (ng/L)	239.6 (87.7)	389.1 (138.4)	<0.0001	202.1 (56.2)	262.4 (96.4)	0.02
Folate (μg/L)	7.9 (4.2)	15.0 (2.7)	<0.0001	3.9 (2.8)	10.6 (5.7)	<0.0001
Vitamin A (μmol/L)	1.2 (0.26)	1.36 (0.28)	0.046	1.0 (0.24)	1.1 (0.31)	0.38
Vitamin E (μmol/L)	29.6 (6.74)	28.9 (5.78)	0.692	31.9 (8.46)	38.1 (7.21)	0.009
Cholesterol (mmol/L)	5.67 (0.82)	5.00 (0.85)	0.007	5.8 (0.79)	6.6 (0.94)	0.011
VitE/Chol	5.26 (1.04)	5.82 (0.91)	0.054	5.5 (1.06)	5.9 (0.86)	0.135
Copper (μmol/L)	32.9 (4.5)	26.1 (4.9)	<0.0001	34.4 (4.1)	28.2 (3.9)	<0.0001
Copper^+^ (μmol/L)	33.1 (4.4)	33.1 (5.3)	<0.0001	34.5 (4.3)	28.0 (3.7)	<0.0001
Zinc (umol/L)	9.7 (1.17)	10.7 (1.30)	0.008	9.1 (0.92)	9.3 (0.94)	0.413
Zinc/Albumin	0.32 (0.04)	0.31 (0.03)	0.172	0.32 (0.02)	0.32 (0.03)	0.958
Selenium (μmol/L)	0.85 (0.13)	1.02 (0.16)	<0.0001	0.79 (0.11)	0.89 (0.14)	0.009
Selenium^+^ (μmol/L)	0.89 (0.14)	1.00 (0.16)	0.050	0.85 (0.11)	0.87 (0.14)	0.437
Total 25OHD (nmol/L)^++^	51.1 (16.89)	68.1 (29.91)	0.070	45.5 (32.54)	68.7 (27.34)	0.093
CRP (mg/L)	12.0 (8.48)	3.7 (3.35)	<0.0001	13.4 (8.1)	4.7 (5.6)	0.001
Albumin (g/L)	30.1 (1.98)	34.5 (2.97)	<0.0001	28.1 (1.2)	29.3 (2.0)	0.034

Data are means (SD). * *P*-value for difference between obese and lean; ^+^ excluding samples with CRP ≥10 mg/L (as inflammatory response known to alter concentrations of these trace elements [18]); ^++^ sufficient sample available for analysis in *n* = 13 lean and *n* = 15 obese in early pregnancy and *n *= 12 lean and *n *= 5 obese in late pregnancy.

There were no significant differences in self-reported total activity levels between very severely obese and lean women in early or late pregnancy (Table 4), but very severely obese women were less likely to take part in vigorous and sporting activity (Table 4). 

**Table 4:** Reported physical activity in early and late gestation in lean and very severely obese women.

Early gestation	Activity levels	Lean *n *= 93			Obese *n *= 148			*P*	*P*^adj^
		Median	C^25^	C^75^	Median	C^25^	C^75^		
Total activity	(MET-h/week)*	17.92	12.11	29.88	23.79	16.23	36.47	0.02	0.21
By intensity	Sedentary activity	11.20	6.73	13.95	10.06	5.56	14.31	0.15	0.34
	Light activity	9.32	5.80	15.05	14.52	8.15	19.35	0.34	0.15
	Moderate activity	8.40	5.05	14.54	9.64	4.76	17.33	0.01	0.24
	Vigorous activity	0.23	0.00	0.94	0.00	0.00	0.23	<0.01	<0.01
By type	Household/care-giving activity	6.20	4.27	15.41	11.50	5.60	20.70	0.46	0.41
	Occupational activity	10.75	9.31	12.53	10.90	0.33	16.04	<0.01	0.09
	Sports/exercise activity	1.93	0.99	3.65	1.13	0.34	2.13	<0.01	<0.01
**Late gestation**	**Activity levels**	**Lean *n *= 87**			**Obese *n *= 146**			***P***	***P*^adj^**
		**Median**	**C^25^**	**C^75^**	**Median**	**C^25^**	**C^75^**		
Total activity	(MET-h/week)*	21.53	13.55	33.37	23.15	14.70	33.02	0.18	0.39
By intensity	Sedentary activity	10.87	7.25	13.82	12.20	7.01	14.01	0.26	0.30
	Light activity	10.66	7.25	13.82	12.97	8.66	19.26	0.17	0.15
	Moderate activity	8.86	4.86	15.76	8.59	4.48	13.98	0.29	0.57
	Vigorous activity	0.23	0.00	0.23	0.00	0.00	0.23	<0.01	<0.01
By type	Household/care-giving activity	7.15	3.37	16.34	10.55	5.94	19.80	0.48	0.26
	Occupational activity	10.97	8.69	15.43	10.97	7.99	15.68	0.25	0.21
	Sports/exercise activity	1.63	0.77	2.88	0.68	0.11	1.99	<0.01	<0.01

C^25^: 25th Percentile, C^75^: 75th Percentile; *P*: unadjusted, *P*^adj^: adjusted for age, parity, ethnicity, DEPCAT and working status; * 1 MET (metabolic equivalent of task) is defined as 1 kcal/kg/h. Total activity excludes sedentary activity.

### 3.3 Adherence to Pregnancy Recommendations

Very severely obese women were more likely to be current smokers but they consumed less alcohol than their lean counterparts (Table 1). Only a third of very severely obese women took folic acid supplements prior to conception compared with 60% in lean; less than one in ten of these women were taking the recommended 5 mg dose. Table 5 shows the percentages of lean and very severely obese women achieving daily recommended nutrient intakes (RNI) during early gestation. Obese women achieved less RNI than lean women. Eighty percent of very severely obese women did not meet the RNI for vitamin K, vitamins D and E, thiamine and fibre; 95% of very severely obese and lean women did not meet the RNI for iron and folate. Overall, only a third of very severely obese (*n* = 37, 35%) and lean (*n* = 22, 31%) women reached the minimum recommended guidelines for physical activity of 16–28 MET h/week during pregnancy. 

**Table 5:** Percentage of lean and obese women achieving recommended nutrient intakes (RNI) in early & late gestation.

Micronutrient	RNI *	Early Gestation				Late Gestation			
		Lean (*n* = 93)		Obese (*n *= 148)		Lean (*n* = 87)		Obese (*n* = 146)	
		%	*n*	%	*n*	%	*n*	%	*n*
Phosphorus	700 mg	100	93	100	148	99	86	97	141
Copper	1 mg	99	92	99	147	100	87	99	144
Manganese	2 mg	99	92	89	132	97	84	14	20
Sodium	1500 mg	98	91	96	142	97	84	95	139
Chloride	2300 mg	98	91	95	141	98	85	95	139
Vitamin C	85 mg	97	90	84	124	91	79	76	111
Vitamin B12	2.6 mg	95	88	92	136	97	84	88	128
Riboflavin	1.4 mg	91	85	79	117	8	7	76	111
Biotin	30 µg	89	83	77	114	91	79	65	95
Magnesium	350 mg	85	79	70	103	77	67	53	77
Vitamin B6	1.9 mg	82	76	80	119	83	72	74	108
Niacin	18 mg	80	74	76	113	78	68	71	104
Calcium	1000 mg	76	71	72	106	77	67	63	92
Zinc	11 mg	67	62	60	89	40	35	48	70
Selenium	60 µg	60	56	53	78	60	52	49	71
PA	6 mg	56	52	60	89	55	48	47	69
Iodine	220 µg	52	48	64	95	61	53	60	87
Vitamin K	90 mg	38	35	18	26	33	29	14	20
Vitamin D	5 µg	33	31	19	28	31	27	19	28
Potassium	4700 mg	25	23	30	44	28	24	32	47
Fibre	28 g	25	23	18	27	21	18	13	19
Vitamin E	15 mg	24	22	22	33	28	24	12	18
Thiamine	1.4 mg	13	12	18	27	86	75	79	116
Folate	600 µg	5	5	5	8	0	0	2	3
Iron	27 mg	4	4	5	8	3	3	2	3

RNI: Recommended nutrient intake; * Based on dietary reference intake guidelines for pregnant women aged 19–50 years (1997–2010). Compiled by the Food and Nutrition Board, Institute of Medicine, Washington.

### 3.4. Dietary Patterns during Pregnancy

Principal component analyses identified two major dietary patterns in early pregnancy among obese women (Table 6) and three major dietary patterns in lean women (Table 7), explaining 66.8% and 75.6% of variance, respectively. Other components explained only 4% or less of the variance. In both groups, the first component was loaded highly across all food groups, and this pattern of eating was labelled “high energy eaters.” This component correlated highly with total energy intake in both groups (obese *r *= 0.94, *p* < 0.0001; lean *r *= 0.92, *p *< 0.0001). The second component in very severely obese (and third component in lean) women showed high loadings across a limited range of food groups and was labelled “select eaters.” In lean women, the second component was loaded highly across fruit and vegetables and low across select other groups and thus was labelled “prudent eaters.” As pregnancy progressed, the “select eater” pattern disappeared in both groups, whilst in the very severely obese a more prudent eating style emerged, with greater consumption of fruit and vegetables similar to that of lean women.

**Table 6:** Patterns of food intake in very severely obese women in early and late pregnancy.

Severely obese women	Early Gestation (*n* = 148)		Late Gestation (*n* = 146)	
Food Intake	Component 1	Component 2	Component 1	Component 2
	“High energy”	“select eater”	“High energy”	“Prudent”
Bread	0.851	0.123	0.869	0.001
Breakfast & Cereals	0.646	0.213	0.747	−0.140
Milk	0.744	0.213	0.774	0.172
Cream & Yogurt	0.594	0.540	0.700	0.308
Cheese	0.742	0.047	0.762	−0.152
Eggs	0.674	−0.381	0.633	−0.196
Meats	0.884	−0.176	0.898	−0.152
Fish	0.751	0.124	0.761	−0.194
Potatoes, Rice & Pasta	0.881	0.042	0.896	−0.116
Savoury foods	0.873	0.037	0.879	0.128
Vegetables	0.814	−0.032	0.808	0.380
Fruit	0.717	0.309	0.777	0.333
Puddings	0.722	−0.215	0.773	−0.015
Chocolates & sweets	0.744	−0.071	0.799	−0.293
Biscuits	0.799	−0.261	0.690	−0.187
Cakes	0.700	−0.484	0.711	−0.218
Spreads & sugars	0.846	−0.060	0.873	−0.075
Soft drinks	0.500	0.208	0.428	0.700

Data show principal component analysis coefficients; higher score indicates greater loading of the food group into the component.

**Table 7:** Patterns of food intake in lean women in early and late pregnancy.

Lean Women	Early Gestation (*n *= 93)			Late Gestation (*n* = 87)	
Food Intake	Component 1	Component 2	Component 3	Component 1	Component 2
	“High energy”	“Prudent”	“Select eater”	“High energy”	“Prudent”
Bread	0.863	−0.029	−0.408	0.897	−0.067
Breakfast & Cereals	0.796	0.008	−0.230	0.770	−0.112
Milk	0.828	0.254	−0.144	0.772	0.311
Cream & Yogurt	0.709	0.017	0.357	0.798	−0.068
Cheese	0.857	−0.128	0.092	0.841	−0.052
Eggs	0.698	−0.315	0.354	0.754	−0.266
Meats	0.841	−0.193	-0.001	0.867	−0.101
Fish	0.797	−0.073	0.299	0.847	−0.053
Potatoes, Rice & Pasta	0.904	0.005	−0.092	0.921	0.019
Savoury foods	0.895	−0.056	0.092	0.901	0.013
Vegetables	0.763	0.301	0.250	0.776	0.303
Fruit	0.882	0.231	−0.004	0.840	0.283
Puddings	0.747	0.023	−0.540	0.767	−0.184
Chocolates & sweets	0.791	−0.087	−0.373	0.799	−0.034
Biscuits	0.743	−0.182	0.192	0.788	−0.076
Cakes	0.767	−0.248	0.284	0.812	−0.289
Spreads & sugars	0.800	−0.025	−0.159	0.893	−0.047
Soft drinks	0.484	0.736	0.242	0.437	0.778

Data show principal component analysis coefficients- higher score indicates greater loading of the food group into the component.

## 4. Discussion 

We found that during pregnancy, very severely obese women consumed diets that were energy-rich but poor in essential micronutrients in comparison to normal-weight controls. Further, in accord with other studies showing that women comply poorly with lifestyle recommendations for pregnancy [20], we found only a third of all women met the recommended levels of physical activity in pregnancy. Very severely obese women, a group at high risk of pregnancy complications, were significantly less likely to adhere to pregnancy lifestyle recommendations than controls, including use of folic acid and smoking habits, suggesting that these are a group in greater need of health behaviour advice prior to and during pregnancy.

The highly inadequate intake of micronutrients in our very severely obese group, which was confirmed by the biochemical analyses, is of particular concern. On average, only 2%–5% of the obese women met the RNIs for iron and folic acid, and less than 20% for vitamins D, E, K and thiamine. Similar patterns have been reported amongst other pregnant populations in the UK [7,21,22,23]. The importance of these micronutrients in influencing pregnancy outcomes is well-recognised, and their deficiency can have a profound effect on development of fetal tissues and organs and also affect long-term infant health status [24]. Poor folic acid intake during pregnancy is one of the maternal risk factors for neural tube defects in babies and may in part explain greater fetal anomaly in this group [25]. Although folic acid intake improved in obese women in the 12 weeks following conception, notably very few were taking the recommended 5 mg dose. Serum folate levels were lower in very severely obese and fell during pregnancy. Both folate and vitamin D deficiencies have been associated with increased risks of pre-eclampsia, preterm delivery, low birth weight and fetal growth restriction [26,27]. Low vitamin D status is also associated with physical anomalies including osteomalacia and skeletal malformations [27]. Iron deficiency is known to cause anaemia during pregnancy [28]. The fact that these detrimental birth outcomes caused by micronutrient deficiency are also closely associated with maternal obesity, deserves attention. The presence of nutritional deficiencies in very severely obese individuals may seem paradoxical in light of the apparent overnutrition state but it has been documented that several micronutrients are deficient in overweight and obese non-pregnant individuals, particularly those who are very severely obese (BMI ≥40 kg/m^2^) such as the women in our study [29]. The cause is not known, but may largely be due to poor quality diet with increased consumption of highly processed foods which are rich in calories but poor in nutrient density, and a proportional reduction of intake of healthier, more nutritious foods [29]. Increased adiposity itself is associated with lower serum levels of some fat-soluble nutrients, such as vitamin D, and this predicts poor status of this nutrient during pregnancy [27]. Likewise concentrations of anti-oxidant micronutrient vitamins A, E and selenium are often lower in smokers [30], perhaps because of increased utilisation, and more of the obese women in our study were current smokers.

There is little published dietary advice for very severely obese pregnant women. Most guidelines are aimed at optimising gestational weight gain rather than focusing on dietary content. We used a principal component analysis to examine dietary patterns, as this may be potentially of greater relevance than nutrients for education of pregnant women about healthy eating. The principal components analysis produced clearly different components in the very severely obese and lean women. In both groups of women, in early pregnancy the first component included those who reported eating foods from most food groups. All coefficients for this component were positive; similar patterns in the literature have been labelled as “high energy” or “high energy-density” [11], and accordingly there was a high correlation of this component with total energy intake. In very severely obese women the second component was a “select eater” group, who ate dairy, carbohydrate and soft drinks but few vegetables. In lean women the second component was a “prudent eater” group, *i.e.,* one with a more balanced diet with greater consumption of fruit and vegetables, in accordance with recommendations from the Department of Health and other agencies. In lean women the “select eater” pattern was the third component. During pregnancy, dietary patterns changed in both groups and the “select eater” pattern disappeared. In the very severely obese group a more “prudent” eating style emerged, similar to the control women. This finding is in contrast to a recent study showing a decrease in diet quality across pregnancy as assessed by the “healthy eating index” in overweight and obese women [31]. This may have been due to the specific healthy eating dietary advice given to very severely obese women attending the clinic. This advice was focused around healthy eating advice using the “eatwell plate” [32] and informing women about portion size.

Physical activity during pregnancy has been widely demonstrated to have various positive effects on women’s health [33]. Current recommendations for exercise in pregnancy are that in the absence of either medical or obstetric complications, all pregnant women should be encouraged to adopt thirty minutes of at least moderate intensity activities for most, if not all, days of the week [34]. We found that only about a third of participants in both of our study groups met the recommended levels of physical activity in pregnancy, findings similar to other studies showing that in general pregnant women do little exercise [35]. Exercising in very severely obese pregnancy may be difficult, particularly if these women are not used to exercising prior to pregnancy. Interventions combined with behavioural change techniques may be required to encourage obese women to exercise in pregnancy [36]. 

Recent guidance from the National Institute of Health and Clinical Excellence in the UK has emphasised a focus on health reinforcement in the periconceptual period [5]. Our results show that greater education regarding smoking, alcohol and folic acid intake amongst women of child-bearing age is required, particularly amongst very severely obese women who are at greater risk of perinatal complications and fetal malformations. The very severely obese women recruited into our study were representative of very severely obese women delivering in the region, other than for parity. Multiparous women were less likely to attend for specialised care, and our sample may also have been a more motivated group, so our findings may have underestimated the adverse health behaviours of very severely obese women. There were more women in our obese group from lower social classes, and so campaigns are needed to reach this high risk group. 

A major strength of our study is the case-control prospective design and the detailed assessment in both early and late pregnancy including biochemical analyses of vitamins and trace elements. In the first trimester of pregnancy, appetite and dietary intake may be lower due to nausea and vomiting, but we did not include women with reported hyperemesis or severe nausea in our analysis. One limitation is that although the food frequency questionnaire describes food intake in the preceding 2–3 months, we did not ask for this information prior to pregnancy. However, it has been demonstrated that dietary patterns change very little from before to the early stages pregnancy [11]. Further, we did not have any information about use of multivitamin supplements prior to or during pregnancy. Another limitation of our study is that the third trimester results (at least in obese) may reflect modification of behaviour (either reported or unreported) in response to advice from the dietician. This may have influenced total calorie intake, and be responsible for attenuation of the differences in energy intake by late gestation between the obese and lean. However, the fact that the obese remained deficient in micronutrients despite advice from a dietician in early pregnancy suggests that our observations may have underestimated the extent of the poor diet in obese pregnant women in the third trimester. The values for median total energy intake and values for macronutrients (protein, fat and carbohydrate), sugars, iron and folic acid in our lean controls were generally comparable to the values reported in other surveys using food frequency questionnaires [21]. Likewise, similar trends of inadequate intake of nutrients such as calcium, iron and folic acid have been reported [23]. 

## 5. Conclusions 

During pregnancy, most women receive close serial medical attention. Motivation is high and many women make lifestyle-changing decisions with potential benefits for their developing child [37], so pregnancy is an ideal time for promotion of healthy behaviour. We did not ask women whether the current pregnancy was planned but it may be that women who plan pregnancy are more likely to adhere to health advice. However, our results clearly demonstrate that more needs to be done to educate women of child-bearing age about the importance of adherence to preconception health advice and that the focus should be on those women in higher risk groups including obesity. The recently published results of two large randomized controlled trials of lifestyle interventions during overweight and obese pregnancy [38,39] showed limited effects on key outcomes such as birthweight despite altering dietary patterns in pregnancy, suggesting that interventions which start prior to pregnancy may be needed. Likewise, the poor nutritional state during pregnancy suggests that specific dietary guidelines with advice tailored for very severely obese women are needed to provide realistic goals that could be maintained during pregnancy and in the long-term.
